# Effect of Viewing Angle on Volume Perceptions for Paired Tumblers

**DOI:** 10.1177/2041669517719296

**Published:** 2017-07-07

**Authors:** Yi-Lang Chen, Yi-Chien Lee, Tzu-Yu Lee, Meng-Zhen Chen

**Affiliations:** Department of Industrial Engineering and Management, Ming Chi University of Technology, Taiwan; Department of Industrial Design, Chang Gung University, Taiwan; Department of Industrial Engineering and Management, Ming Chi University of Technology, Taiwan

**Keywords:** volume perception, viewing angle, tumbler characteristics, visual cue

## Abstract

This study examined how tumbler characteristics influenced the perception of volume at different viewing angles. Three tumbler characteristics were individually examined, namely, shape, size, and elongation. At four viewing angles (0°, 30°, 60°, and 90°), 50 participants poured a certain amount of liquid (150 or 200 mL) into a designated tumbler according to their perception. Results showed that tumbler size and elongation influenced volume perception. At viewing angles of 0° and 30°, the participants poured more liquid into short-wide tumblers than into tall-slender tumblers. At viewing angles of 60° and 90°, the results were opposite. The reason may be that the change of viewing angle made the participant’s sight cues from the container diameter more visible than those from the container height. Similar results were obtained for the pair of small and large tumblers. However, no effect of viewing angle on tumblers with different geometric shapes was observed. The contradictory results in comparison with those of previous studies may be related to viewing angle; in addition, the effect of viewing angle was also influenced by the characteristics of tumblers.

## Introduction

Optical illusion is an interesting topic. When considering two straight lines with identical length (one vertical line and one horizontal line), the vertical line appears longer than the horizontal line (the horizontal–vertical illusion). Coren and Girgus (1978) indicated that the Delboeuf illusion (identified 150 years ago) has been regarded as robust, but “of little practical value.” This means that although these optical illusions are noteworthy, they are only for academic research on psychological operations and are of little practical value.

In recent years, these optical illusions have gradually received attention and have been applied in daily life and product design. A series of studies revealed that people overestimated the volume of tall, slender tumblers and underestimated the volume of short, wide tumblers (Wansink & van Ittersum, 2003, 2005). A previous study on obese children in a 6-week health and fitness camp had found that because of underestimating the volume of short, wide tumblers, children drank 74% more juice (Wansink & van Ittersum, 2003). In addition, experienced bartenders’ glass volume perceptual errors also reached 26% (Wansink & van Ittersum, 2005). Fifty years ago, studies on the ratio of the height to diameter of tumblers (elongation) were conducted (Been, Braunstein, & Piazza, 1964; Pearson, 1964). The well-known developmental psychologist Piaget (1968) found that children often used liquid height in a container to estimate liquid volume and neglected the diameter of the container. Raghubir and Krishna (1999) investigated the perceived volume of tall, slender as well as short, wide tumblers and obtained similar results. Wansink, van Ittersum, and Painter (2006) used the horizontal–vertical illusion to explain the perceived tumbler volume. In other words, when a person used a short, wide tumbler, the person would unconsciously pour more wine or other beverages (Wansink & van Ittersum, 2005).

Previous researchers have also applied visual illusions in studies on crockery type and perceived volume. Wansink and Cheney (2012) reported that, when a round dish was used to hold food, the Delboeuf illusion would cause a person to perceive a larger amount of food contained in a smaller round dish, therefore increasing food intake by 56%. Some studies have also explored how dish color influenced perception of volume (van Ittersum & Wansink, 2012); the results of which have been extensively applied to catering, pharmacy, and nutrition. For example, home spoons should not be used as a container to give children medicine (Wansink & van Ittersum, 2010), and dieters should choose their cutlery carefully (Raghubir & Krishna, 1999). Kerr, Patterson, Koenen, and Greenfield (2009) found that pub customers’ estimates of wineglass volume were not influenced by shape; however, wineglass size significantly influenced perceived volume. Large wine tumblers caused people to pour more wine.

Lan (2008) and Chen and Shi (2016) conducted studies similar to that of Wansink and van Ittersum (2005), but used opaque tumblers and sitting postures, respectively; they obtained results opposite to those of Wansink and van Ittersum (2005). This may be because participants perceive liquid volume differently from various viewing angles. In the study of Wansink and van Ittersum (2005), the participants had sufficient visual cues regarding liquid height to judge liquid volume. In the studies of Lan (2008) and Chen and Shi (2016), an angle of top view and a sitting posture were individually adopted, and liquid volume was judged on the basis of cup diameter. The viewing angle used in the experiments of Lan (2008) and Chen and Shi (2016) differed from that used in the study by Wansink and van Ittersum. Accordingly, in terms of the participant’s viewpoint, cues from the container diameter were more visible than were those from the container height, and opposite results were obtained.

Recently, Szocs and Lefebvre (2017) investigated the effect of viewing angle on consumers’ portion size perceptions and consumption. They found that when participants had a downward viewing angle, they perceived the portion as larger when it was presented horizontally. They concluded that, when individuals view a plate of food at a downward angle (e.g., when seated at a dining table) the surface area is easier to encode than the height dimension and therefore is used as a heuristic for size. The finding from the study of Szocs and Lefebvre (2017) may partially explain the contradictory results between that of Lan (2008) and Chen and Shi (2016) and the previous studies. However, only surface area perceptions of a plate of foods (not the tumblers) were examined by Szocs and Lefebvre (2017). In the present study, we, therefore, systematically explored the differences between perceived volume and actual volume of various types of tumblers from various viewing angles and compared these with the results obtained in previous studies. The hypothesis of this study was that participants might differently perceive the liquid volumes poured into the paired tumblers because different visual cues would dominate when pouring liquid at four viewing angles. In this study, three tumbler pairs with different characteristics, namely shape, size, and elongation, were examined.

## Methods

### Participants

Fifty participants were recruited in this study. The age of the participants was between 18 and 28 years (men: *n* = 25, mean age = 22.6 years, *SD* = 2.3; women: *n* = 25, mean age = 23.0 years, *SD* = 2.1). The participants were undergraduate or graduate students. No participant exhibited visual defects such as color blindness or color weakness. The inclusion criteria were no vision defect with the naked eye or after vision correction. Informed consent was obtained from all participants, and the experimental procedure was approved by the Ethics Committee of Chang Gung Memorial Hospital.

### Paired Tumblers

**Figure 1.**
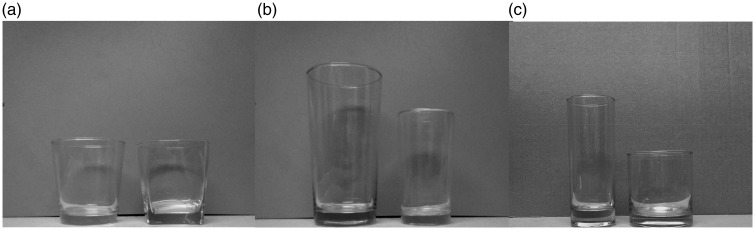
Three pairs of tumblers with various characteristics used in the present study. (a) Geometric shape (round and square); (b) Identical ratio of tumbler diameter to tumbler height (large and small); (c) Elongation (tall, slender tumbler and short, wide tumbler).

The tumblers used in this study are shown in Figure 1. We chose tumblers that would be familiar to our Taiwanese participants; these were purchased from a hypermarket and included various pairs with varying geometric shape, size, and elongation. Table 1 showed the characteristics of the tumblers used in the study. The maximum volumes of the tumblers were equal to 230 mL, except in the size manipulation condition in which ratio of small to large tumblers was 2/3 (i.e., 250 vs. 370 mL). The liquid volumes poured for the paired tumblers of shape and elongation characteristics in the experiment were set at 150 mL, which were approximately 65% of their maximum volumes. The volumes poured into the small and large tumblers were set at 80.0% and 54.1% of the maximum volumes, respectively. The averaged poured volume was also set at appropriately 65% of the total maximum volumes.

**Table 1.** Maximum Volumes and the Poured Volumes of the Paired Tumblers for Various Tumbler Characteristics.

Tumbler characteristics	Paired tumblers	Maximum volumes (mL)	Poured volumes (mL)	% of the maximum volumes for pouring
Shape	Round	230	150	65.2
	Square	230	150	65.2
Size	Large	370	200	54.1
	Small	250	200	80.0
Elongation	Tall-slender	230	150	65.2
	Short-wide	230	150	65.2

### Experimental Design

**Figure 2.**
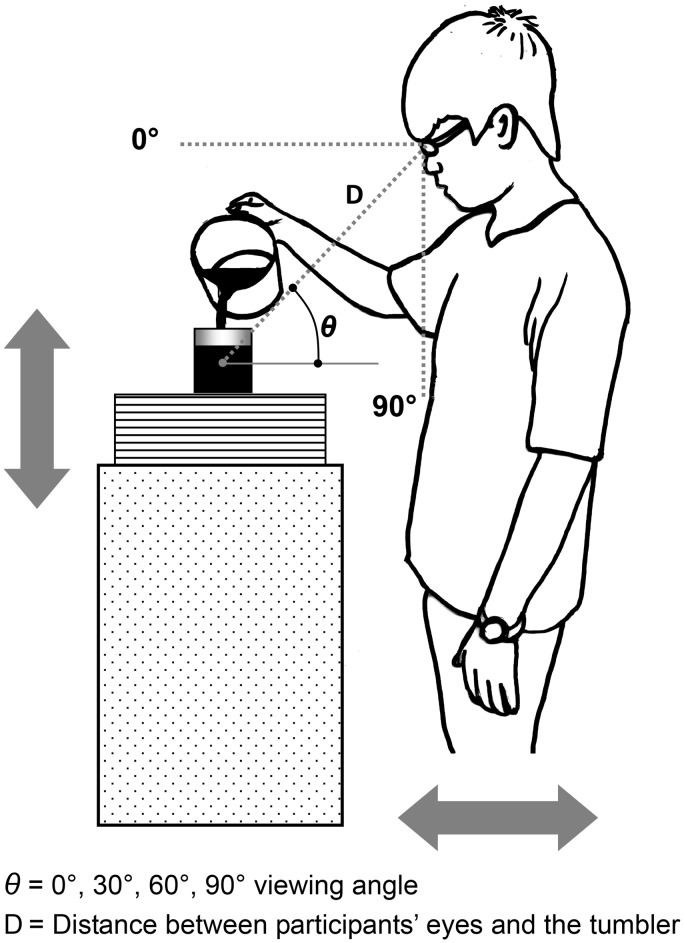
The experimental setting of the liquid pouring test.

In this study, three characteristics of tumbler (i.e., shape, size, and elongation) were separately considered (Figure 1). The liquid volume perceptions between the pairs of each characteristic were compared; however, the three tumbler characteristics were not mutually compared because of different characteristic conditions and poured liquid volumes (150 or 200 mL). For each tumbler pair, four viewing angles (0°, 30°, 60°, and 90°) were also examined. As a result, 400 estimates of volume were collected (i.e., 50 participants × 2 tumblers (each pair) × 4 viewing angles) for each characteristic of paired tumblers. In the experiment, the counterbalanced method was employed (Attwood, Scott-Samuel, Stothart, & Munafò, 2012). For example, when using paired tumblers, the first participant was first required to pour 200 mL of liquid into a large tumbler and was then required to pour 200 mL of liquid into a small tumbler; the next participant was first required to pour 200 mL liquid into a small tumbler and was then required to pour 200 mL liquid into a large tumbler. The sequences of three characteristics of tumblers and four viewing angles were arranged in a random order. The viewing angle used in the experiment should be based on a standing posture, and was defined as the angle formed by the line connecting the eyes of participants to the geometric center of tumbler shape and the horizontal line (Figure 2). In the present study, wood veneers (wood veneers were 1-cm thick) were used to construct platforms for the tumblers at different heights to establish various viewing angles. The platform height was adjusted by subtracting or adding the wood veneers to keep the designated viewing angle for each participant. To reduce the influence of distances on participants’ judgments, for the four viewing angles, the distance between the center of a tumbler and the eyes of a participant was kept constant (45 cm). Moreover, participants were also requested to move horizontally along the sagittal plane if necessary. To reduce the variance of viewing angles, we employed a sagittal plane camera to monitor and correct the participants’ viewing angles and the distance between the participants’ eyes and tumblers.

### Procedures

Following Wansink and van Ittersum (2005) and Lan (2008), a liquid pouring procedure was employed in the study. For various viewing angles, the participants stood naturally and avoided excessive head movements. Before the experiment began, we explained the experimental procedures to the participants and measured the height of the participants’ eyes from the ground to determine viewing angles. Moreover, we provided the participants with a round tumbler (100 mL) which was irrelevant to the experimental tumblers to practice for 5 min and to be familiar with the perception of liquid volume. The experimental data were then collected at least 1 hr after the participant practice period. Subsequently, the experimenter requested the participants to pour liquid (150 or 200 mL) from a 500-mL water bottle (Figure 2) to a designated tumbler at a designated viewing angle. If the participants felt they poured excessive liquid, they could use a straw to remove the liquid. Once the participant determined the poured volume, the experimenter weighed the liquid in the tumbler and recorded the liquid volume. On the basis of previous experiments, the water was colored to give it the appearance of juice or coke.

### Data Analysis

In the experiment, we collected data on the liquid volume poured into various containers at various viewing angles. In this study, three separate tests for each paired tumblers (i.e., shape, size, elongation) were independently considered. A two-way repeated-measures analysis of variance (ANOVA) was therefore used to investigate the effects of each paired tumblers for four viewing angles. Moreover, a one-way ANOVA was then carried out individually by each paired tumblers at each viewing angle and individually by each tumbler type at four viewing angles; that is, a total of 18 one-way ANOVA procedures were conducted. In the analysis, Bonferroni correction was used for post hoc comparisons. A statistical package, Statistical Product and Service Solutions (SPSS, version 19.0), was used for data analysis. The significance level for ANOVA was set at 0.05, and for Bonferroni correction was set at 0.004 (i.e., 0.05/12) for two-way ANOVA and 0.008 (i.e., 0.05/6) for one-way ANOVA for multiple comparisons when the effect of viewing angle variable was significant in the analysis.

## Results and Discussion

Table 2 shows the two-way ANOVA results for perceived volumes by three tumbler pairs. Viewing angle significantly affected the poured volumes in different tumbler sizes (*p* < .001), whereas no effect was found in other tumbler pairs. This implies that the effect of the viewing angle on volume perception may differ among the various tumbler characteristics. Further analysis was performed for tumbler size condition using Bonferroni correction and found that difference in poured liquid volumes only existed between the viewing angles at 0° and 90° (with a difference of 15.6 mL) when averaged across the poured volumes of large and small tumblers (*p* < .004).

**Table 2.** Two-Way ANOVA Results for Each of Three Tumbler Characteristics.

Variables	*df*	SS	MS	*F* (*df*, 392)	*p*	Power
View angle	3	2962	988	1.53	.208	0.402
Shape	1	388	388	0.59	.440	0.120
View angle × Shape	3	354	118	0.19	.909	0.084
View angle	3	23598	7866	7.23	<.001	0.983
Size	1	121	121	0.11	.739	0.063
View angle × Size	3	36000	12000	11.07	<.001	0.999
View angle	3	521	174	0.27	.845	0.102
Elongation	1	91	91	0.14	.706	0.066
View angle × Elongation	3	21716	7239	11.42	<.001	0.999

*Note.* Shape: round vs. square; Size: large vs. small; Elongation: tall-slender vs. short-wide.

**Figure 3.**
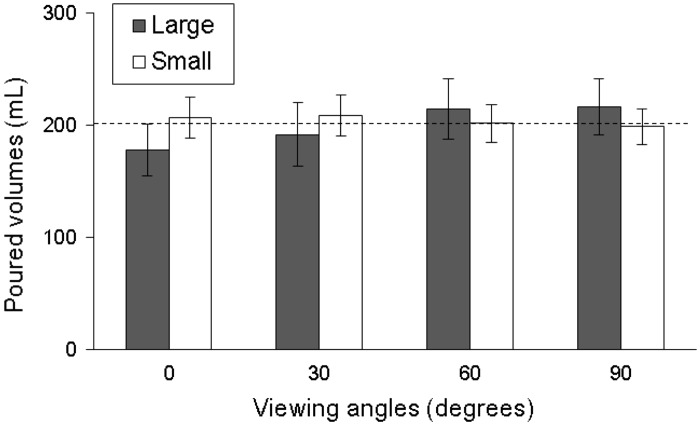
The comparison of the poured liquid volumes (data in mean ± standard deviation) between the large and small tumblers at four viewing angles (the dashed line means the target volume, 200 mL).

**Figure 4.**
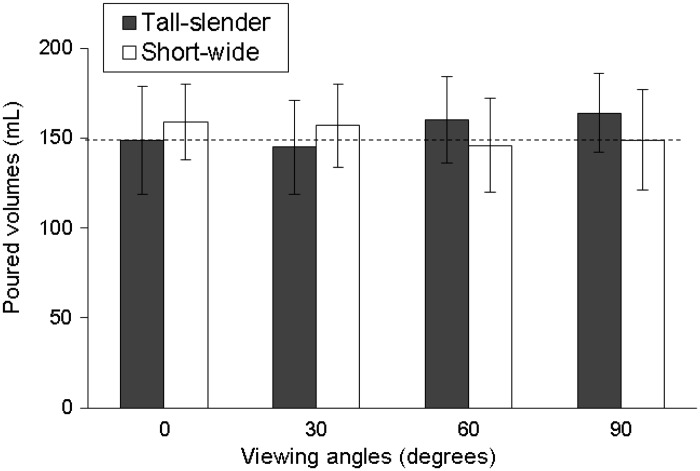
The comparison of the poured liquid volumes (data in mean ± standard deviation) between the tall-slender and short-wide tumblers at four viewing angles (the dashed line means the target volume, 150 mL).

It is worth noting that, as shown in Table 2, the effect of two-way interaction (viewing angle × tumbler variable) was significant on liquid volume poured in tumbler pairs of size and elongation (all *p* < .001). Figures 3 and 4 illustrate the results. The figures show that at various viewing angles, the participants perceived the volume of tumbler pairs that differed by size and elongation inconsistently. At viewing angles of 0° and 30°, the participants poured less liquid in large and tall-slender tumblers than in small and short-wide tumblers. A viewing angles of 60° and 90°, the results were the opposite. One-way ANOVA results (Table 3) further show that differences in liquid volumes poured between two size tumblers at 0° and 30° were −29.3 and −16.6 mL, respectively; differences in liquid volumes poured between two elongation tumblers at 0° and 30° were −9.4 and −11.7 mL, respectively. For the size effect, difference in liquid volumes was 13.0 mL at 60° and 18.5 mL at 90°. For the elongation effect, difference in liquid volumes was 14.5 mL at 60° and 14.4 mL at 90° (all *p* < .05). As shown in Table 3, viewing angle showed a significant effect on volume perception when pouring liquid into the large tumbler (*p* < .001), tall-slender tumbler (*p* < .001), and short-wide tumbler (*p* < .05), whereas other tumbler types revealed no significant effect. Further multiple comparisons using the Bonferroni correction showed that significant differences in the effect of viewing angle on volume perceptions were observed in large tumbler (0° vs. 60°, 0° vs. 90°, and 30° vs. 90°) and tall-slender tumbler (0° vs. 90° and 30° vs. 90°) (all *p* < .008). However, there was no significance observed in the short-wide tumbler. This means that not only the tumbler pairs but also the single tumbler type may directly influence the volume perception by different viewing angles. However, in this study, the tumbler shape did not significantly influence the perception of volume under four viewing angles (all *p* > .05, Table 3).

**Table 3.** Results of One-Way ANOVA for the Volume Perceptions Examined by Each Paired Tumblers at Each Viewing Angle and by Each Tumbler Type at Four Viewing Angles.

View angles (°)	Mean (SD) poured volumes (mL)		*F* (1, 98)	*p*
	Round tumbler	Square tumbler		
0	156.9 (27.6)	157.1 (29.5)	0.00	.942
30	151.6 (27.0)	150.8 (25.5)	0.02	.764
60	152.8 (23.3)	148.1 (25.5)	3.82	.051
90	152.0 (23.2)	149.4 (21.0)	0.94	.334
*F* (3, 196)	1.23	0.47		
*p*	*p* = .299	*p* = .707		
	Large tumbler	Small tumbler		
0	177.5 (22.9)	206.8 (18.4)	17.42	<.001***
30	192.3 (27.7)	208.9 (17.8)	7.62	.006**
60	214.6 (26.6)	201.6 (16.7)	7.25	.007**
90	217.0 (25.6)	198.5 (16.1)	7.77	.005**
*F* (3, 196)	15.68	0.27		
*p*	*p* < .001***	*p* = .847		
	Tall-slender tumbler	Short-wide tumbler		
0	149.3 (30.3)	158.7 (21.2)	6.51	.011*
30	145.6 (26.5)	157.3 (23.6)	11.07	<.001***
60	160.2 (24.6)	145.7 (26.1)	12.74	<.001***
90	163.5 (21.5)	149.1 (28.1)	11.86	<.001***
*F* (3, 196)	6.73	3.04		
*p*	*p* < .001***	*p* = .029*		

* *p* < .05. **p < .01. ***p < .001.

According to previous studies, tumbler shape (Raghubir & Krishna, 1999), tumbler size (Kerr et al., 2009), and tumbler elongation (Wansink & van Ittersum, 2005) resulted in differences in volume perception. However, previous studies presented different results because of various experimental protocols and tumblers used for the experiments, as well as racial and ethnic differences (Kerr et al., 2009). In the present study, we first investigated how characteristics of tumblers (shape, size, and elongation) influenced the perception of volume at various viewing angles. We observed a viewing angle effect and that various viewing angles might have opposite effects. The results of this study show that our hypothesis was verified in the tumbler pairs of size and elongation.

**Figure 5.**
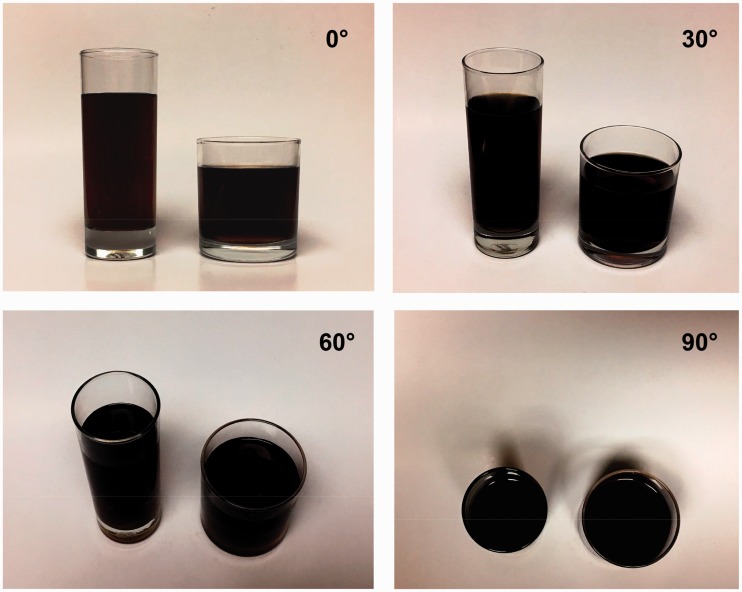
Visual cues at four viewing angles (using a tall, slender tumbler and a shorter, wide tumbler as an example).

Regarding the influence of the tumbler elongation on the perception of volume, Wansink and van Ittersum (2005) found that because of the horizontal–vertical illusion, people poured more wine into short, wide tumblers than they did into tall, slender tumblers; in their study, the participants had sufficient cues about liquid height (i.e., the viewing angle was nearly 0°). In contrast to the study of Wansink and Ittersum (2005), Lan (2008) used opaque tumblers for the experiment and found that when the cue about liquid height became unavailable, the participants judged volume on the basis of a top-view perspective; accordingly, the cue used to judge volume was the size of the cup diameter. This is the reason that Lan (2008) obtained results opposite to those of Wansink and van Ittersum (2005) which the horizontal–vertical illusion was speculated. Chen and Shi (2016) requested 90 participants to adopt a sitting posture and perform a test; they used transparent tumblers for their experiment and obtained the same results as Lan’s study (2008). Szocs and Lefebvre (2017) found that when individuals view a plate of food at a downward angle (e.g., when seated at a dining table), the surface area is easier to encode than the height dimension and therefore is used as a heuristic for size, resulting in larger food portion perceived. In the present study, we used viewing angles from 0° to 90°, and covered all viewing angles used in previous studies. When we used more horizontal viewing angles (0° and 30°), we obtained the same results as Wansink and van Ittersum (2005); when we used more vertical viewing angles (60° and 90°), we obtained results similar to that of Lan (2008) and Chen and Shi (2016). Our study showed that when considering the elongation effect, viewing angles might have caused the contradictory results with the previous studies; in other words, different viewing angles may cause different visual cues and thus affect the participants’ perception (Figure 5).

The present study also investigated a viewing angle effect for large and small tumblers. At the viewing angle of 90°, the participants poured more juice into large tumblers than they did into small tumblers. In other words, when the same amount of juice is poured into large and small tumblers, the participants perceived less juice volumes in large tumblers than they did in small tumblers; thus, the participants tended to pour more juice into large tumblers to obtain an identical perception of juice volume for large and small tumblers. Kerr et al. (2009) conducted a study in a pub and found that participants poured more wine into large tumblers; the results accorded with the present study. In this study, when the viewing angles were at 0° and 30°, the participants poured more wine into small tumblers than they did into large tumblers; different viewing angles caused opposite results. In the study of Kerr et al. (2009), the participants were customers and thus should have adopted a nearly top-view perspective (Chen & Shi, 2016). Their results accorded with the present study, indicating that for small and large tumblers, viewing angle might influence the amount of liquid poured into tumblers. However, the results of this study showed that complex factors (more than a single factor) influenced the amount of liquid poured into tumblers, such as testing protocol. For example, in the study, the target amount (200 mL) for the large tumbler was relatively lower (54% of the maximum volume), while it was much closer to full for the small tumbler (80% of the maximum volume) and resulted in a more accurate response in the small tumbler than in the large one. This may cause the relatively unchangeable liquid volumes poured into the small tumbler among four viewing angles (in a range of approximately 10 mL, Figure 3). This may also partially explain why the main effect of viewing angle variable was significant in size manipulation condition but nonsignificant in elongation manipulation condition. However, the two-way interaction effects on volume perceptions for the two tumbler characteristics (i.e., size and elongation) were significant (Table 2).

In the study, tumbler shape and viewing angle had no effect on the amount of liquid poured into round or square tumblers (maximum volume, 230 mL). To our knowledge, no systematic studies on the influence of tumbler geometric shape on the amount of liquid poured into the tumblers have been conducted. In the present study, tumblers used in previous studies were employed; the causal relationship among variables was complex. This study preliminarily used two geometric shapes (round shape vs. square shape) and controlled other variables and found that viewing angle did not influence the amount of liquid poured into tumblers. Future studies can explore the effects of other tumbler shapes (e.g., pyramidal and cylindrical shapes).

As aforementioned, the findings of this study imply that not only the tumbler pairs but also the single tumbler type may influence the volume perception by different viewing angle. The result was unexpected. This study limitation is a confounding factor and merits further clarification. In the experiment where tumbler size and elongation were considered, viewing angle individually influenced the amount of liquid poured into tumblers; these phenomena likely occurred because of different visual cues. According to the results, significant differences in the effect of viewing angle were observed between relatively horizontal viewing angles (0° and 30°) and vertical viewing angles (60° and 90°) for the large tumbler and the tall-slender tumbler, indicating that the threshold for the change of the dominant visual cue for these tumblers should be between 30° and 60°. Whether the threshold is approximately 45° remains to be investigated.

## Conclusion

In the present study, 50 participants were recruited for a juice pouring experiment to examine the effect of viewing angles on the volume perceptions of tumblers. The results showed that the contradictory results with previous studies might be due to participants’ viewing angles during liquid pouring. The findings suggest that the effect of the tumbler characteristics on volume perception may depend on various viewing angles; in addition, the effect of viewing angles was also influenced by the characteristics of the tumblers.
